# Supplementary material for: A Systematic Review on Clinical and Health-Related Quality of Life Outcomes following Total Gastrectomy in Patients with Hereditary Diffuse Gastric Cancer
Source: Cancers (Basel). 2024 Jan 23;16(3):473. doi: 10.3390/cancers16030473 (PMC10854827; doi:10.3390/cancers16030473)
Supplement: Supplementary file 1 [file cancers-16-00473-s001.zip › cancers-2670370-supplementary.pdf]

Supplementary Materials

Table S1: Search Strategy.

PubMed (3/3/2023)

Search	Query	Hits
#5	#4 Limit: Publication Date from 2000 to 2022	60
#4	#3 Limit: Full-text English articles	
#3	#1 AND #2	81
#2	"CDH1 mutation or pathogenic variant or germline mutation [MeSH Major Topic]" OR "CDH1 mutation or pathogenic variant or germline mutation [MeSH Subheading]"	1, 031
#1	"CDH1 pathogenic variants [MeSH Major Topic]" OR "Hereditary diffuse gastric cancer [MeSH Subheading]" including "prophylactic total gastrectomy", "surgical outcomes", "surgery", "complications", "histopathology", "pathology", "health-related quality of life"	1, 135

Medline (4/3/2023)

Search	Query	Hits
#3	#2 Limit: Publication Date from 2000 to 2022	45
#2	#1 Limit: Full-text English articles	
#1	CDH1 pathogenic variants OR CDH1 mutation OR Hereditary diffuse gastric cancer AND [[Prophylactic total gastrectomy or gastrectomy] OR [surgical out-comes] OR surgery OR histopathology OR health-related quality of life OR quality of life]	78

Scopus (6/3/2023)

Search	Query	Hits
#5	#4 Limit: Publication Date from 2000 to 2022	68
#4	#3 Limit: Full-text English articles	
#3	#1 AND #2	78
#2	[Prophylactic total gastrectomy or gastrectomy] OR [surgical outcomes] OR surgery OR histopathology OR health-related quality of life OR quality of life	202 54
#1	CDH1 pathogenic variants OR CDH1 mutation OR Hereditary diffuse gastric cancer	20298

Cochrane Library (6/3/2023)

Search	Query	Hits
#5	#4 Limit: Publication Date from 2000 to 2022	55
#4	#3 Limit: Full-text English articles	
#3	#1 AND #2	68
#2	"Prophylactic total gastrectomy or gastrectomy [MeSH Major Subheading] OR "Surgical outcomes [MeSH Major Subheading]" OR "Surgery [MeSH Major Subheading]" OR "Histopathology [MeSH Major Subheading] OR "Health-related quality of life [MeSH Major Subheading]" OR "Quality of life [MeSH Major Subheading]"	1, 135
#1	"CDH1 pathogenic variants [MeSH Major Topic]" OR " Hereditary dif-fuse gastric cancer [MeSH Major Topic]" OR " Hereditary dif-fuse gastric cancer [MeSH Major Topic]"	238

Table S2: Newcastle-Ottawa Quality Assessment Scale for Included Studies.

**Item**[1]	**Reference No.**																			
	**13**	**14**	**15**	**16**	**17**	**18**	**19**	**20**	**21**	**22**	**23**	**24**	**25**	**26**	**27**	**28**	**29**	**30**	**31**	**32**
**Selection**																				
Representativeness of the exposed cohort	*	*	*	*	*		*			*	*	*	*	*	*		*	*	*	*
Selection of non-exposed cohort	*		*	*		*	*	*	*	*	*	*	*	*	*	*		*	*	*
Ascertainment of exposure	*	*	*	*	*	*	*	*	*	*	*		*	*	*	*	*	*	*	*
Demonstration that outcome of interest was not present at start of study	*	*	*	*	*					*	*	*	*		*	*	*	*		
**Comparability**																				
Comparability of cohorts on the basis of the design or analysis		*		*		*		*			*		*	*	*	*	*		*	*
**Outcome**																				
Assessment of outcome	*	*	-	*			*	*	*	*	*	*	*	*	*	*	*	*	*	*
Was follow-up long enough for outcomes to occur	*	*	*		*	*			*	*	*		*	*	*	*				
Adequacy of follow-up of cohorts				*	*	*	*	*		*	*	*	*	*			*	*	*	*

[1] A study can be awarded a maximum of one star for each numbered item within the “Selection” and “Exposure” categories. A maximum of two stars can be given for the “Comparability” category. A higher number of stars indicate higher quality studies.

Table S3: Complications following Total Gastrectomy in Included Studies.

Type of Complication																
**No.**	**Total number of patients (n)**	**Anastomotic stricture n (%)**	**Anastomotic leak n (%)**	**Duodenal stump leak n (%)**	**Gastrointestinal bleeding n (%)**	**Ileus n (%)**	**Intussusception n (%)**	**Multiple organ system failure n (%)**	**Pulmonary complication n (%)**	**Venous thromboembolism/ Pulmonary embolism n (%)**	**Septic phlebitis n (%)**	**Small bowel obstruction n (%)**	**Urinary tract infection n (%)**	**Wound infection n (%)**	**Others n (%)**	**Reference**
1.	23		2 (8.7%)	-	-	2 (8.7%)	-	-	2 (8.7%)	3 (13.0%)	-	2 (8.7%)	2 (8.7%)	4 (17.4%)	-	14
2.	10	1 (10.0%)	-	-	-	-	1 (10.0%)	-	1 (10.0%)	-	-	1 (10.0%)	-	-	-	15
3.	6	1 (16.7%)	-	-	-	-	-	-	-	-	-	1 (16.7%)	-	-	-	17
4.	11	-	2 (18.2%)	-	-	-	-	-	2 (18.2%)	-	-	-	-	-	-	19
5.	41	-	3 (7.3%)	2 (4.9%)	2 (4.9%)	-	-	1 (2.4%)	6 (14.6%)	1 (2.4%)	-	-	1 (2.4%)	3 (7.3%)	-	20
6.	26	-	2 (7.7%)	-	-	3 (11.5%)	-	-	1 (3.8%)	-	-	-	-	-	3 (11.5%)	21
7.	101	5 (5.0%)	6 (6.0%)	1 (1.0%)	2 (2.0%)	-	-	-	2 (2.0%)	2 (2.0%)	-	-	1 (1.0%)	8 (8.0%)	-	22
8.	19	-	-	-	1 (5.3%)	-	-	-	1 (5.3%)	-	-	-	-	-	-	23
9.	10	-	1 (10.0%)	-	1 (10.0%)	-	-	-	1 (10.0%)	-	-	-	-	-	-	24

10.	54	-	-	-	-	-	-	-	-	1 (1.9%)			1 (1.9%)	2 (3.7%)	1 (3.7%)	27
**Overall complication rate (%)**																
	353	7 (2.0%)	16 (4.5%)	3 (0.8%)	6 (1.7%)	5 (1.4%)	1 (0.3%)	1 (0.3%)	16 (4.5%)	7 (2.0%)	-	4 (1.1%)	5 (1.4%)	17 (4.8%)	3 (0.8%)	13-27
